# Coping changes the brain

**DOI:** 10.3389/fnbeh.2013.00013

**Published:** 2013-02-22

**Authors:** Jordan M. Nechvatal, David M. Lyons

**Affiliations:** ^1^Department of Psychiatry and Behavioral Sciences, Stanford University School of MedicineStanford, CA, USA; ^2^Neurosciences Program, Stanford University School of MedicineStanford, CA, USA

**Keywords:** stress, coping, exposure therapy, neuroplasticity, neuroimaging, learning, emotion regulation

## Abstract

One of the earliest and most consistent findings in behavioral neuroscience research is that learning changes the brain. Here we consider how learning as an aspect of coping in the context of stress exposure induces neuroadaptations that enhance emotion regulation and resilience. A systematic review of the literature identified 15 brain imaging studies in which humans with specific phobias or post-traumatic stress disorder (PTSD) were randomized to stress exposure therapies that diminished subsequent indications of anxiety. Most of these studies focused on functional changes in the amygdala and anterior corticolimbic brain circuits that control cognitive, motivational, and emotional aspects of physiology and behavior. Corresponding structural brain changes and the timing, frequency, and duration of stress exposure required to modify brain functions remain to be elucidated in future research. These studies will advance our understanding of coping as a learning process and provide mechanistic insights for the development of new interventions that promote stress coping skills.

## Introduction

Diverse therapeutic and preventive interventions utilize intermittent stress exposure to enhance the development of stress coping skills. Intermittent exposure to stress is, for example, an aspect of resiliency training for people that work in conditions where performance in the face of adversity is required, e.g., medical and military personnel, aviators, police, firefighters, and rescue workers (Meichenbaum, 2007; Stetz et al., 2007). Exposure therapy for anxiety disorders likewise teaches patients to imagine a graded series of stress-inducing objects or situations, and then encourages interaction with these stressors *in vivo*. These procedures promote learning and provide opportunities to practice stress coping skills (Tryon, 2005; McNally, 2007; Craske et al., 2008). Here we consider how learning as an aspect of coping in the context of stress exposure therapy changes the brain. This perspective builds on evidence that learning reflects experience-dependent neuroadaptations in brain regions that mediate cognitive, motivational, and emotional aspects of physiology and behavior (Poldrack, 2000; Posner and DiGirolamo, 2000; Dolan, 2002; Pascual-Leone et al., 2005).

Initially, we conducted a *PUBMED* (www.ncbi.nlm.nih.gov/pubmed) search using the terms “exposure therapy” AND “brain” to identify 49 published reports. Excluding review papers and empirical studies of animal models, obsessive compulsive disorder, and interventions not related to exposure reduced the search results to eight reports. Seven additional studies cited in the initial eight reports were subsequently found to be appropriate for inclusion in the review, bringing the total to 15 studies listed in Table 1. Treatment efficacy was established in all 15 studies based on behavioral outcomes not addressed in the summaries presented below except for relevant brain-behavior correlations identified by various neuroimaging techniques. Nine of the 15 studies examined patients with specific phobias and six studies examined patients with post-traumatic stress disorder (PTSD). Seven of the nine studies of phobias focused on fear of spiders and we begin by reviewing these reports in chronological order.

**Table 1:** **List of studies in the review**.

**Citation**	**Disorder**	**Exposure therapy duration**	**Brain imaging modality**	**Interval between scans**	**Preplanned brain regions**	**Exploratory brain regions**
Paquette et al., 2003	Spider phobia	Multiple sessions 4 weeks	fMRI	6 weeks	PFC^*^	Frontal gyrus, fusiform gyrus, occipital gyrus, parahippocampal gyrus, parietal cortex
Straube et al., 2006	Spider phobia	Multiple sessions 2 days, 4–5 h/day	fMRI	2 weeks	Amygdala, ACC^*^, fusiform gyrus^*^, insula^*^, parahippocampal gyrus, PFC^*^, thalamus^*^	Basal ganglia, central gyrus, cuneus, frontal gyrus, lingual gyrus, occipital gyrus, parietal gyrus, precentral gyrus, precuneus, temporal gyrus
Goossens et al., 2007	Spider phobia	Single session 4–5 h	fMRI	2 weeks	Amygdala^*^, ACC^*^, insula^*^	Occipital cortex
Schienle et al., 2007	Spider phobia	Single session 4 h	fMRI	2 weeks	Amygdala, ACC, fusiform gyrus, insula^*^, OFC^*^, parahippocampal gyrus, PFC	Angular gyrus, frontal gyrus, lingual gyrus, occipital gyrus, parietal gyrus, supramarginal gyrus
Leutgeb et al., 2009	Spider phobia	Single session 4 h	EEG	1 week	n/a	n/a
Leutgeb et al., 2012	Spider phobia	Single session 4 h	EEG	1 week	n/a	n/a
Hauner et al., 2012	Spider phobia	Single session 2–3 h	fMRI	2 hours/week/6 months follow up	Amygdala^*^	ACC, frontal gyrus, fusiform gyrus, insula, lingual gyrus, occipital cortex, parietal lobe, PFC, temporal gyrus
Nave et al., 2012	Snake phobia	Single session 2–3 h	fMRI	2 weeks	Amygdala, ACC, frontal gyrus^*^, insula, OFC	n/a
Furmark et al., 2002	Public speaking phobia	Multiple sessions 9 weeks	PET	9 weeks	Amygdala^*^, hippocampus^*^	Temporal cortex
Lindauer et al., 2005	PTSD	Multiple sessions 4 months	sMRI	4 months	Amygdala, hippocampus, parahippocampal gyrus	n/a
Felmingham et al., 2007	PTSD	Multiple sessions 8 weeks	fMRI	6 months	Amygdala, ACC^*^	Frontal gyrus, hippocampus, parietotemporal gyrus, postcentral gyrus, temporal gyrus
Rabe et al., 2008	PTSD	Multiple sessions 8–12 weeks	EEG	3 months	n/a	n/a
Lindauer et al., 2008	PTSD	Multiple sessions 4 months	SPECT	4 months	n/a	Frontal gyrus
Roy et al., 2010	PTSD	Multiple sessions 6 weeks	fMRI	2 months	Amygdala^*^, ACC, hippocampus	Frontal gyrus, PFC, subcallosal gyrus
Adenauer et al., 2011	PTSD	Multiple sessions 12 weeks	MEG	4 months	n/a	Parietal cortex, occipital cortex

*Brain regions are specified for preplanned comparisons and post-hoc exploratory analyses. Asterisks signify a statistically significant exposure therapy treatment effect for the preplanned comparisons*.

Abbreviations *ACC* *anterior cingulate cortex* *OFC* *orbitofrontal cortex* *PFC* *prefrontal cortex* *PTSD* *post-traumatic stress disorder* *EEG* *electroencephalography* *fMRI* *functional magnetic resonance imaging* *PET* *positron emission tomography* *sMRI* *structural magnetic resonance imaging* *SPECT* *single-photon emission computed tomography* *n/a* *not applicable.*

## Spider phobia

Paquette et al. (2003) compared healthy non-phobic controls (*n* = 13) with spider phobics (*n* = 12) before and after exposure therapy in a functional magnetic resonance imaging (fMRI) study of blood oxygenation as a measure of neural activity. Exposure therapy entailed guided mastery training, education for correcting spider misbeliefs, and exposure to pictures of spiders, films of spiders, and real spiders. During the initial scan session and prior to therapy, responses to spiders vs. butterflies were greater in dorsolateral prefrontal cortex and the parahippocampal gyrus in spider phobics compared to non-phobic controls. After exposure therapy, responses to spiders were diminished in right dorsolateral prefrontal cortex and right parahippocampal gyrus compared to pre-treatment fMRI data. These findings suggest that exposure therapy results in decreased demands on brain regions that mediate cognitive strategies involved in self-regulation (prefrontal cortex) and de-conditioning of traumatic memories (hippocampus).

Straube et al. (2006) randomized patients with spider phobia to exposure therapy (*n* = 14) or a wait list control condition (*n* = 14), and compared these patients to healthy non-phobic controls (*n* = 14) using fMRI. Exposure therapy entailed training to enhance cognitive restructuring and exposure to pictures of spiders, touching tarantula spider skin, and handling real spiders. Before exposure therapy, responses to spiders vs. neutral pictures were greater in the anterior cingulate cortex, insula, and left extrastriate visual cortex of phobic patients compared to non-phobic controls. Conversely, non-phobic controls showed greater responses in the left amygdala, bilateral parahippocampal gyrus, and pre- and post-central gyri. After exposure therapy, responses to spiders were diminished in the anterior cingulate cortex, insula, left dorsal medial prefrontal cortex, thalamus, and left precuneus in the exposure therapy group compared to wait list controls. The exposure therapy group also exhibited greater responses in the right cuneus relative to wait list controls. Within-subjects comparisons confirmed post-treatment reductions in the response to spiders in anterior cingulate cortex, insula, and left thalamus. Non-phobic controls were not scanned twice so their responses from the initial scan session were compared to second scan responses for the exposure therapy and wait list groups. No significant differences were observed between non-phobic controls and the exposure therapy group after treatment, but the wait list group continued to exhibit greater activity in the anterior cingulate cortex and right insula compared to healthy controls. These results together suggest normalized anterior cingulate and insula activity in phobic patients successfully treated with exposure therapy.

Goossens et al. (2007) examined patients with spider phobia (*n* = 16) before and after exposure therapy, and compared these patients to healthy non-phobic controls (*n* = 14) using fMRI. Exposure therapy entailed education for correcting spider misbeliefs and exposure to drawings of spiders, photographs of spiders, and real spiders. During the initial scan session and prior to therapy, responses to spiders vs. neutral pictures were greater in the left amygdala, anterior cingulate cortex, and insula in phobics compared to non-phobic controls. After exposure therapy, group differences were no longer discerned in the amygdala, anterior cingulate cortex, or insula. Within-subjects comparisons confirmed post-treatment reductions in the left amygdala, anterior cingulate cortex, and insula. Furthermore, percent change in the amygdala response to spiders vs. neutral pictures positively correlated with therapy-related outcome measures of fear and anxiety. These findings support earlier suggestions that exposure therapy normalizes the activity of corticolimbic brain circuits.

Schienle et al. (2007) randomized patients with spider phobia to exposure therapy (*n* = 14) or a wait list control condition (*n* = 12), and compared these patients to healthy non-phobic controls (*n* = 25) using fMRI. Exposure therapy consisted of a single 4-h session of progressive gradual approach tasks involving a live spider. During the initial scan session and prior to therapy, greater responses to spiders vs. neutral pictures were discerned in the left amygdala and fusiform gyrus for phobic patients compared to non-phobic controls. Conversely, non-phobic controls exhibited greater activity in the right inferior parietal gyrus, inferior frontal gyrus, anterior cingulate, medial orbitofrontal cortex, and right dorsolateral prefrontal cortex compared to phobic subjects. After exposure therapy, greater responses to spiders were discerned in bilateral medial orbitofrontal cortex in successfully treated patients compared to wait list controls. Within-subjects comparisons confirmed post-treatment right orbital frontal increases and also revealed reduced activity in the right insula. Additional analyses revealed a positive correlation between the reduction of experienced anxiety and somatic panic symptoms and decreased activation in the right amygdala and left insula. The reduction of arousal ratings was also positively correlated with a decreased right amygdala response.

In a follow-up study using the same experimental design, Schienle and colleagues randomized patients with spider phobia to exposure therapy (*n* = 22) or a wait list control condition (*n* = 23), and compared these patients with healthy non-phobic controls (*n* = 20) in an electroencephalography (EEG) study (Leutgeb et al., 2009). Event related potentials were extracted for three well characterized and temporally precise time windows following spider vs. neutral stimulus onset. Before exposure therapy, larger parietal P300 and early-late positive potential (early-LPP) amplitudes were discerned in response to spiders vs. neutral pictures in phobics compared to non-phobic controls. After exposure therapy, greater central late-late positive potential (late-LPP) amplitudes were discerned in response to spiders for successfully treated patients compared to wait list controls. Parietal P300 and early-LPP amplitudes remained unchanged from pre-treatment levels. Schienle and colleagues found similar LPP enhancement in a recent EEG study of children with spider phobia (Leutgeb et al., 2012) and suggest that exposure therapy alters neural markers of attention allocation by reducing attentional avoidance and changing the way spiders are perceived.

Hauner et al. (2012) examined 12 patients with spider phobia before and after exposure therapy using fMRI. Exposure therapy entailed a 14-step series of progressive approach tasks with a live tarantula spider conducted during a single 2–3 h session. During the initial scan session and prior to therapy, responses to spiders vs. neutral pictures were greater in right amygdala, anterior cingulate cortex, insula, and ventral medial prefrontal cortex, while diminished responses were discerned in right dorsolateral prefrontal cortex. After successful treatment, self-reported reductions in fear induced by spider pictures were accompanied by increased activity in right dorsolateral prefrontal cortex with decreased activity in amygdala, anterior cingulate cortex, insula, and ventral medial prefrontal cortex. Additionally, increased responses to spiders vs. neutral pictures were observed immediately after treatment in right superior parietal cortex.

Six months after treatment, Hauner et al. (2012) found that in patients free from phobic symptoms responses to spiders were similar to those observed immediately after exposure therapy for amygdala and limbic brain regions. However, dorsolateral prefrontal cortex responses were diminished relative to responses observed immediately after exposure therapy, and diminished responses were also discerned in bilateral ventral visual cortex. These findings indicate that brain changes can occur long after completion of exposure therapy and suggest a time-limited role for top-down prefrontal control of amygdala and limbic brain systems.

## Snake phobia

Nave et al. (2012) conducted a double-blind placebo controlled trial of exposure therapy combined with D-cycloserine in patients with snake phobia using fMRI. Patients were randomized to placebo plus exposure therapy (*n* = 9) or D-cycloserine plus exposure therapy (*n* = 7) to determine whether D-cycloserine enhances treatment efficacy. Exposure therapy entailed a 13-steps series of progressive gradual approach tasks involving a live snake. After exposure therapy, responses to snakes vs. neutral pictures were diminished in right dorsal prefrontal cortex in both the D-cycloserine and placebo groups compared to pre-treatment fMRI data. Other prefrontal brain regions showed qualitatively different responses in the exposure therapy plus D-cycloserine vs. placebo conditions but the significance of these findings is not obvious because patients in both treatment conditions showed equivalent reductions in snake phobia severity. In the placebo group, additional analyses revealed a positive correlation between the reduction of snake phobia severity and decreased snake-elicited activation in right/middle frontal gyrus, right insula/inferior frontal gyrus, bilateral medial orbitofrontal cortex, left orbitofrontal cortex, and bilateral amygdala. In the D-cycloserine group, affective ratings scores were negatively correlated with perigenual cingulate cortex activation and positively correlated with left ventrolateral prefrontal cortex activation.

## Public speaking anxiety

In a study of public speaking anxiety by Furmark et al. (2002), patients were randomized to exposure therapy (*n* = 6), citalopram medication (*n* = 6), or a wait list control condition (*n* = 6). Exposure therapy entailed training to enhance cognitive restructuring, homework assignments, and interaction with stressful simulated public speaking situations. All patients were scanned using positron emission tomography (PET) with oxygen 15-labeled H_2_0 as an indicator of regional cerebral blood flow (rCBF) while presenting a short speech to a 6–8 person audience. After exposure therapy or the citalopram treatments, rCBF responses to public speaking were diminished in the amygdala, hippocampus, and anterior and medial temporal cortex compared to pre-treatment PET data. Pre- vs. post-treatment changes in rCBF were not observed in the wait list control condition. Between-group comparisons confirmed that rCBF reductions occurred in both treatment conditions compared to the wait list control condition, with particularly strong responses discerned in right temporal lobe regions. Additionally, favorable outcomes at 1-year follow-up were associated with greater initial suppression of the subcortical rCBF response to public speaking in the periaqueductal gray area, left thalamus, and bilateral amygdala. These results suggest that both pharmacologic and exposure-based therapies may act through a common mechanism to attenuate amygdalar-limbic hyperactivity and diminish public speaking anxiety.

## Post-traumatic stress disorder

In addition to serving as an effective treatment for phobic disorders, exposure therapy is often used to treat patients with PTSD. In the only structural brain imaging study that met our search criteria, Lindauer et al. (2005) investigated structural brain changes in patients with PTSD related to personal violence or exposure to accidents or disasters randomized to exposure therapy (*n* = 9) or a wait list control condition (*n* = 9). These patients were compared to trauma-experienced, but non-PTSD controls (*n* = 14) using structural MRI to measure the volume of specific brain regions. Before exposure therapy, PTSD patients had significantly smaller total, right, and left hippocampal volumes, and larger total and left parahippocampal gyrus volumes compared to traumatized, non-PTSD controls corrected for total brain volume variation. Volumetric changes were not discerned in any brain region after exposure therapy for PTSD.

Felmingham et al. (2007) examined eight patients with personal assault or motor vehicle accident-related PTSD before and after exposure therapy using fMRI. Imaginal exposure was combined with training to enhance cognitive restructuring. Before exposure therapy, responses were greater in right postcentral gyrus, right middle temporal gyrus, and left superior temporal gyrus in response to fearful vs. neutral stimuli. After exposure therapy, responses to the same fearful stimuli were increased in bilateral anterior cingulate cortex, left middle temporal gyrus, right inferior frontal gyrus, left parietotemporal gyrus, and right hippocampus. Post-treatment increases in anterior cingulate cortex activity were positively correlated with symptom improvements, whereas amygdala activity was negatively correlated with symptom improvements.

Rabe et al. (2008) examined patients with motor vehicle accident-related PTSD or subsyndromal PTSD randomized to exposure therapy (*n* = 17) or a wait list control condition (*n* = 18) using EEG. Imaginal exposure, writing exposure, and *in vivo* exposure were combined with cognitive restructuring education and relaxation training. After exposure therapy, diminished right anterior brain responses to trauma-related vs. neutral pictures were discerned in treated patients compared to wait list controls. Right anterior brain responses were correlated with outcome measures of PTSD severity.

In a follow-up to their initial brain structural imaging study described above, Lindauer et al. (2008) randomized patients with PTSD related to personal violence or exposure to accidents or disasters to exposure therapy (*n* = 10) or a wait list control condition (*n* = 10), and compared these patients to trauma-experienced but non-PTSD controls (*n* = 15) using single-photon emission computed tomography (SPECT) to measure rCBF. Imaginal exposure was combined with psychoeducation, writing tasks, and training to enhance cognitive restructuring. Before exposure therapy, greater rCBF responses were discerned in the right insula and right dorsolateral prefrontal cortex in PTSD patients compared to traumatized non-PTSD controls. After exposure therapy, decreased rCBF responses to trauma-related imaginal exposure were discerned in right dorsolateral prefrontal cortex in treated patients compared to wait list controls. The effect of treatment on outcome measures of PTSD severity correlated positively with rCBF changes in the left superior temporal gyrus and middle frontal gyrus.

Roy et al. (2010) examined military service members with PTSD randomized to either imaginal (*n* = 8) or virtual reality (*n* = 7) exposure therapy using fMRI. Imaginal exposure involved imaginary recall of the traumatic experience in progressively greater detail, while virtual reality exposure included interaction with trauma-relevant virtual visual environments with multi-sensory stimulation, i.e., tactile vibrations and the smell of cordite during virtual explosions. Due to high rates of dropout and resulting small sample sizes, imaginal vs. virtual reality therapy could not be compared. By collapsing across these two treatment conditions, a diminished post-treatment amygdala response to negative, but not neutral, stimuli on the Affective Stroop Test was discerned relative to pre-treatment data.

Adenauer et al. (2011) randomized PTSD patients to exposure therapy (*n* = 16) or a wait list control condition (*n* = 18) in a magnetoencephalography (MEG) study. Due to subject dropout and other exclusion criteria, post-treatment MEG data analysis included 11 exposure therapy subjects and eight wait list controls. Exposure therapy entailed a narrative analysis focused on the detailed reconstruction of each patient's traumatic memory. During the initial scan session and prior to therapy, no group differences were discerned in cortical responses to aversive vs. neutral pictures. After exposure therapy, greater responses were discerned in superior parietal cortex for treated patients compared to wait list controls. Additionally, within-subjects comparisons revealed greater left occipital cortical responses to aversive vs. neutral stimuli after exposure therapy compared to pre-treatment MEG data. Adenauer and colleagues discuss these findings as evidence that exposure therapy decreases attentional avoidance and enhances voluntary control over previously avoided traumatic memories.

## Functional brain changes

All but one study that met our search criteria used functional brain imaging modalities to examine how stress exposure changes the brain. Four of the 14 functional studies used EEG or MEG, which both have high temporal resolution but are spatially restricted to cortical surface potentials spanning 1–2 cm. The remaining 10 functional studies used PET, SPECT, or fMRI, which all have limited temporal resolution but are able to target both cortical and deeper brain structures with millimeter spatial resolution.

The temporal precision of MEG and EEG allows for the characterization and dissociation of attentional brain network processes that occur within milliseconds of stimulus presentation. Two relevant event-related potentials, the P300 and the LPP, have been shown to reflect increased attention toward motivationally relevant stimuli. The P300 peaks between 300 and 500 ms following stimulus onset and has been linked to automatic attention processes, while the LPP typically peaks between 500 and 3000 ms of stimulus onset and has been associated with controlled attention and emotion regulation (Olofsson et al., 2008; Dunning and Hajcak, 2009). The spider phobia EEG studies report enhanced LPP amplitudes and no change in P300 amplitudes in response to spiders in treated patients (Leutgeb et al., 2009, 2012). The stability of P300 amplitudes suggests that exposure therapy does not change automatic attention processes. Enhancement of LPP amplitudes likely reflects reduced attentional avoidance to arousing stimuli as patients learn through repeated exposures that feared consequences do not occur and that avoidance is unnecessary for anxiety reduction. Adenauer et al. (2011) similarly attribute their MEG findings to brain mechanisms involved in reducing attentional avoidance.

In the functional studies based on PET, SPECT, and fMRI, preplanned comparisons and exploratory analyses converged on five regions of interest, i.e., amygdala, prefrontal cortex, anterior cingulate cortex, insula, and hippocampus (Table 2). All of these regions are known to be involved in emotion regulation and resilience. In studies of healthy humans, for example, cognitive efforts aimed at reducing negative emotions decrease amygdala responses determined by fMRI (Ochsner et al., 2002, 2004). Exposure therapy likewise diminished amygdala responses in four different studies (Furmark et al., 2002; Goossens et al., 2007; Roy et al., 2010; Hauner et al., 2012) and none of the studies reported a post-treatment increase (Table 2). Four studies also consistently noted exposure therapy-induced down regulation of the insula (Straube et al., 2006; Goossens et al., 2007; Schienle et al., 2007; Hauner et al., 2012). This region integrates somatic signals for interoceptive awareness and participates in networks needed to determine stimulus salience and attentional focus (Paulus and Stein, 2006).

**Table 2:** **Summary of functional brain changes**.

**Citation**	**Disorder**	**Amygdala**	**ACC**	**Hipc**	**Insula**	**PFC**
Paquette et al., 2003	Spider phobia	–	–	↓	–	↓
Straube et al., 2006	Spider phobia	–	↓	–	↓	↓
Goossens et al., 2007	Spider phobia	↓	↓	–	↓	–
Schienle et al., 2007	Spider phobia	–	–	–	↓	↑
Hauner et al., 2012	Spider phobia	↓	↓	–	↓	↑↓
Nave et al., 2012	Snake phobia	–	–	–	–	↓
Furmark et al., 2002	Public speaking phobia	↓	–	↓	–	–
Felmingham et al., 2007	PTSD	–	↑	↑	–	↑
Lindauer et al., 2008	PTSD	–	–	–	–	↓
Roy et al., 2010	PTSD	↓	–	–	–	–

*Preplanned comparisons and exploratory analyses converged on five most common regions of interest in the functional studies based on PET, SPECT, and fMRI. Post-treatment increases (↑), decreases (↓), or no-change (–) in functional activity are shown for each region*.

Abbreviations *ACC* *anterior cingulate cortex* *Hipc* *hippocampus* *PFC* *prefrontal cortical regions* *PTSD* *post-traumatic stress disorder.*

Prefrontal cortex plays a key role in top-down control of amygdala activity and thereby regulates behavioral responses to emotionally salient events (Salzman and Fusi, 2010; Todd et al., 2011). In animal models, prefrontal control of amygdala activity mediates learned extinction of conditioned fear (Gottfried and Dolan, 2004; Delgado et al., 2006), and fMRI studies of humans have identified inverse correlations between increased prefrontal and decreased amygdala responses to emotional stimuli (Ochsner et al., 2002, 2004). In this review, we found only a single study that specifically reported increased prefrontal and decreased amygdala responses to emotional stimuli after stress exposure therapy (Hauner et al., 2012). Furthermore, five of seven studies (Table 2) that identified exposure therapy-induced changes in prefrontal activity actually reported post-treatment decreases in prefrontal responses (Paquette et al., 2003; Straube et al., 2006; Lindauer et al., 2008; Hauner et al., 2012; Nave et al., 2012).

Additional inconsistencies were evident in anterior cingulate cortex and hippocampus (Table 2). Three studies reported exposure therapy-induced down regulation of anterior cingulate cortex activity (Straube et al., 2006; Goossens et al., 2007; Hauner et al., 2012) in keeping with its role in assessing the salience of emotional information (Etkin et al., 2011), but one study noted post-treatment increases anterior cingulate cortex responses (Felmingham et al., 2007). Two studies reported that exposure therapy decreases hippocampal responses to subsequent presentations of emotional stimuli (Furmark et al., 2002; Paquette et al., 2003), but one study reported increased post-treatment hippocampal responses (Felmingham et al., 2007).

One possible explanation for the discrepancies noted above is that exposure therapy may have different neurobiological effects in patients with different specific phobias or patients with PTSD related to warfare trauma vs. motor vehicle accidents. Inconsistencies may also arise from differences between studies in the duration of exposure therapy which ranged from single sessions spanning a few hours to multiple sessions spanning many weeks (Table 1). Likewise, variation in the content and type of stress exposure during each exposure session may contribute to inconsistent results as demands on specific brain regions will vary in a task-specific manner. Additionally, many studies incorporate various neutral stimuli from the International Affective Picture System but no clearly neutral standard yet exists. Brain responses to spiders vs. butterflies, for example, are likely to differ from responses to spiders vs. other “neutral” stimuli such as snails, household items, or geometric objects.

Discrepancies in functional brain changes may also arise from differences in the timing of brain scans relative to the exposure therapy sessions (Table 1). Exposure therapy effects are dynamic and may emerge with varying temporal delays, and resulting brain changes may be long-lasting or transient depending on the specific brain functions affected. For example, dorsolateral prefrontal cortex responses to spider stimuli were increased immediately after exposure therapy in the fMRI study by Hauner et al. (2012), but 6 months later the same brain region showed a diminished response to the same spider stimuli. The investigators indicate that the initial post-treatment brain scan but not the 6-months follow up scan overlapped with exposure-induced engagement of corticolimbic regions that were transiently involved in learning and memory consolidation.

## Structural brain changes

Despite evidence that brain functions rely on structural scaffolding and communication across distributed networks of neurons (Mesulam, 1990; Kolb and Whishaw, 1998; Citri and Malenka, 2008; Singer, 2009) we found only a single study designed to determine whether stress exposure therapy induces structural changes in corticolimbic brain circuits (Lindauer et al., 2008). Stress exposure therapy effects were not detected in this study but other forms of learning are known to induce structural changes in the brain (Gaser and Schlaug, 2003; Draganski et al., 2004; Zatorre et al., 2012). Remarkably, these changes can occur rapidly as only a few min of practicing a whole-body balancing task increases gray matter volumes in frontal and parietal brain regions with gray matter expansion maintained for up to several weeks (Taubert et al., 2010).

Experience-dependent brain changes have also been reported for white matter tissue determined by diffusion tensor imaging (Bengtsson et al., 2005; Scholz et al., 2009). Diffusion tensor imaging measures diffusion-driven displacements of water molecules. Water diffusion is less restricted in the direction of white matter axon bundles than in the perpendicular direction, and measures of this anisotropy can be used to characterize microstructural properties that are sensitive to alterations in white matter myelination and axonal integrity (Le Bihan, 2003, 2006). As an insulator of axons, myelin modifies nerve conduction velocities and, in turn, increases or decreases the functional synchrony of organized neural networks (Szeligo and Leblond, 1977; Salami et al., 2003; Yamazaki et al., 2010). Future investigations of brain changes induced by stress exposure therapy may therefore benefit by combining functional and structural brain imaging modalities to determine whether functional outcomes reflect structural changes and vice versa.

## Limitations

All studies have their limitations and our analysis of stress coping-induced brain changes is no exception. Each of the 15 studies reviewed here exposed patients to stressful conditions that, on average, diminished subsequent measures of anxiety. Coping in the context of stress exposure therapy was further promoted in several of the studies by professional training to enhance cognitive restructuring, relaxation, guided mastery, and diverse forms of psychoeducation. The extent to which specific training procedures modify the neurobiological effects of concomitant exposure to stress remains to be determined.

Additional studies are needed to assess whether stress coping-induced brain changes spontaneously occur in healthy humans as well as patients with disorders beyond those considered here. Nine of the 15 studies examined patients with specific phobias and the remaining six studies examined patients with PTSD. Seven of the nine studies of phobias focused on fear of spiders. Despite the potentially promising extension of principles derived from exposure therapy to explain empirically supported treatments for depression (Tryon and Misurell, 2008), we failed to find a published study of exposure therapy-related brain changes in this patient population.

Sample sizes are limited in all 15 studies and high dropout rates are prevalent in the studies of PTSD. Additionally, some patients with PTSD were comorbid for depression and/or were taking medications. Several of the studies compared brain imaging data from patients scanned both before and after exposure therapy with data acquired during single scan sessions for healthy controls. Patient post-treatment comparisons with controls are therefore confounded with potential test/re-test effects. The various brain imaging modalities considered in our review differ in their strengths and limitations as summarized above. The simultaneous application and integration of diverse brain imaging modalities within a single study may provide a more comprehensive understanding of stress coping-induced changes in the brain.

## Summary and conclusions

In summary, the literature on brain changes induced by learning as an aspect of coping in the context of stress exposure therapy highlights functional neuroadaptations in brain regions that mediate emotion regulation and resilience. Corresponding structural brain changes and the duration, frequency, and timing of stress exposure required to modify brain functions now remain to be elucidated in detail. Such studies will provide mechanistic insights for the development of new interventions that enhance the adaptive aspects of coping with stress. The neuroscience of coping is an untapped resource for the development of new interventions because little is known about the neurobiology of learning to cope with stress.

### Conflict of interest statement

The authors declare that the research was conducted in the absence of any commercial or financial relationships that could be construed as a potential conflict of interest.

## References

[B1] Adenauer H. Catani C. Gola H. Keil J. Ruf M. Schauer M. (2011). Narrative exposure therapy for PTSD increases top-down processing of aversive stimuli – evidence from a randomized controlled treatment trial. BMC Neurosci. 12:127 10.1186/1471-2202-12-127 22182346 PMC3258226

[B2] Bengtsson S. L. Nagy Z. Skare S. Forsman L. Forssberg H. Ullén F. (2005). Extensive piano practicing has regionally specific effects on white matter development. Nat. Neurosci. 8, 1148–1150 10.1038/nn1516 16116456

[B3] Citri A. Malenka R. C. (2008). Synaptic plasticity: multiple forms, functions, and mechanisms. Neuropsychopharmacology 33, 18–41 10.1038/sj.npp.1301559 17728696

[B4] Craske M. G. Kircanski K. Zelikowsky M. Mystkowski J. Chowdhury N. Baker A. (2008). Optimizing inhibitory learning during exposure therapy. Behav. Res. Ther. 46, 5–27 10.1016/j.brat.2007.10.003 18005936

[B5] Delgado M. R. Olsson A. Phelps E. A. (2006). Extending animal models of fear conditioning to humans. Biol. Psychol. 73, 39–48 10.1016/j.biopsycho.2006.01.006 16472906

[B6] Dolan R. J. (2002). Emotion, cognition, and behavior. Science 298, 1191–1194 10.1126/science.1076358 12424363

[B7] Draganski B. Gaser C. Busch V. Schuierer G. Bogdahn U. May A. (2004). Neuroplasticity: changes in grey matter induced by training. Nature 427, 311–312 10.1038/427311a 14737157

[B8] Dunning J. P. Hajcak G. (2009). See no evil: directing visual attention within unpleasant images modulates the electrocortical response. Psychophysiology 46, 28–33 10.1111/j.1469-8986.2008.00723.x 18992071

[B9] Etkin A. Egner T. Kalisch R. (2011). Emotional processing in anterior cingulate and medial prefrontal cortex. Trends Cogn. Sci. 15, 85–93 10.1016/j.tics.2010.11.004 21167765 PMC3035157

[B10] Felmingham K. Kemp A. Williams L. Das P. Hughes G. Peduto A. (2007). Changes in anterior cingulate and amygdala after cognitive behavior therapy of posttraumatic stress disorder. Psychol. Sci. 18, 127–129 10.1111/j.1467-9280.2007.01860.x 17425531

[B11] Furmark T. Tillfors M. Marteinsdottir I. Fischer H. Pissiota A. Långström B. (2002). Common changes in cerebral blood flow in patients with social phobia treated with citalopram or cognitive-behavioral therapy. Arch. Gen. Psychiatry 59, 425–433 10.1001/archpsyc.59.5.425 11982446

[B12] Gaser C. Schlaug G. (2003). Brain structures differ between musicians and non-musicians. J. Neurosci. 23, 9240–9245 14534258 10.1523/JNEUROSCI.23-27-09240.2003 PMC6740845

[B13] Goossens L. Sunaert S. Peeters R. Griez E. J. L. Schruers K. R. J. (2007). Amygdala hyperfunction in phobic fear normalizes after exposure. Biol. Psychiatry 62, 1119–1125 10.1016/j.biopsych.2007.04.024 17706612

[B14] Gottfried J. A. Dolan R. J. (2004). Human orbitofrontal cortex mediates extinction learning while accessing conditioned representations of value. Nat. Neurosci. 7, 1144–1152 10.1038/nn1314 15361879

[B15] Hauner K. K. Mineka S. Voss J. L. Paller K. A. (2012). Exposure therapy triggers lasting reorganization of neural fear processing. Proc. Natl. Acad. Sci. U.S.A. 109, 9203–9208 10.1073/pnas.1205242109 22623532 PMC3384187

[B16] Kolb B. Whishaw I. Q. (1998). Brain plasticity and behavior. Annu. Rev. Psychol. 49, 43–64 10.1146/annurev.psych.49.1.43 9496621

[B17] Le Bihan D. (2003). Looking into the functional architecture of the brain with diffusion MRI. Nat. Rev. Neurosci. 4, 469–480 10.1038/nrn1119 12778119

[B18] Le Bihan D. (2006). [From Brownian motion to mind imaging: diffusion MRI]. Bull. Acad. Natl. Méd. 190, 1605–1627 discussion: 1627. 17650747

[B19] Leutgeb V. Schäfer A. Köchel A. Schienle A. (2012). Exposure therapy leads to enhanced late frontal positivity in 8- to 13-year-old spider phobic girls. Biol. Psychol. 90, 97–104 10.1016/j.biopsycho.2012.02.008 22388043 PMC3330248

[B20] Leutgeb V. Schäfer A. Schienle A. (2009). An event-related potential study on exposure therapy for patients suffering from spider phobia. Biol. Psychol. 82, 293–300 10.1016/j.biopsycho.2009.09.003 19751797

[B21] Lindauer R. J. L. Booij J. Habraken J. B. A. van Meijel E. P. M. Uylings H. B. M. Olff M. (2008). Effects of psychotherapy on regional cerebral blood flow during trauma imagery in patients with post-traumatic stress disorder: a randomized clinical trial. Psychol. Med. 38, 543–554 10.1017/S0033291707001432 17803835

[B22] Lindauer R. J. L. Vlieger E. J. Jalink M. Olff M. Carlier I. V. E. Majoie C. B. L. M. (2005). Effects of psychotherapy on hippocampal volume in out-patients with post-traumatic stress disorder: a MRI investigation. Psychol. Med. 35, 1421–1431 10.1017/S0033291705005246 16164766

[B23] McNally R. J. (2007). Mechanisms of exposure therapy: how neuroscience can improve psychological treatments for anxiety disorders. Clin. Psychol. Rev. 27, 750–759 10.1016/j.cpr.2007.01.003 17292521

[B24] Meichenbaum D. (2007). Stress inoculation training: a preventative and treatment approach, in Principles and Practice of Stress Management, 3rd Edn., eds Lehrer P. M. Woolfolk R. L. Sime W. E. (New York, NY: Guilford Press), 497–518

[B25] Mesulam M. M. (1990). Large-scale neurocognitive networks and distributed processing for attention, language, and memory. Ann. Neurol. 28, 597–613 10.1002/ana.410280502 2260847

[B26] Nave A. M. Tolin D. F. Stevens M. C. (2012). Exposure therapy, d-cycloserine, and functional magnetic resonance imaging in patients with snake phobia. J. Clin. Psychiatry 73, 1179–1186 10.4088/JCP.11m07564 23059145

[B27] Ochsner K. N. Bunge S. A. Gross J. J. Gabrieli J. D. E. (2002). Rethinking feelings: an FMRI study of the cognitive regulation of emotion. J. Cogn. Neurosci. 14, 1215–1229 10.1162/089892902760807212 12495527

[B28] Ochsner K. N. Ray R. D. Cooper J. C. Robertson E. R. Chopra S. Gabrieli J. D. E. (2004). For better or for worse: neural systems supporting the cognitive down- and up-regulation of negative emotion. Neuroimage 23, 483–499 10.1016/j.neuroimage.2004.06.030 15488398

[B29] Olofsson J. K. Nordin S. Sequeira H. Polich J. (2008). Affective picture processing: an integrative review of ERP findings. Biol. Psychol. 77, 247–265 10.1016/j.biopsycho.2007.11.006 18164800 PMC2443061

[B30] Paquette V. Lévesque J. Mensour B. Leroux J. M. Beaudoin G. Bourgouin P. (2003). “Change the mind and you change the brain”: effects of cognitive-behavioral therapy on the neural correlates of spider phobia. Neuroimage 18, 401–409 10.1016/S1053-8119(02)00030-7 12595193

[B31] Pascual-Leone A. Amedi A. Fregni F. Merabet L. B. (2005). The plastic human brain cortex. Annu. Rev. Neurosci. 28, 377–401 10.1146/annurev.neuro.27.070203.144216 16022601

[B32] Paulus M. P. Stein M. B. (2006). An insular view of anxiety. Biol. Psychiatry 60, 383–387 10.1016/j.biopsych.2006.03.042 16780813

[B33] Poldrack R. A. (2000). Imaging brain plasticity: conceptual and methodological issues— a theoretical review. Neuroimage 12, 1–13 10.1006/nimg.2000.0596 10875897

[B34] Posner M. I. DiGirolamo G. J. (2000). Cognitive neuroscience: origins and promise. Psychol. Bull. 126, 873–889 11107880 10.1037/0033-2909.126.6.873

[B35] Rabe S. Zoellner T. Beauducel A. Maercker A. Karl A. (2008). Changes in brain electrical activity after cognitive behavioral therapy for posttraumatic stress disorder in patients injured in motor vehicle accidents. Psychosom. Med. 70, 13–19 10.1097/PSY.0b013e31815aa325 17991819

[B36] Roy M. J. Francis J. Friedlander J. Banks-Williams L. Lande R. G. Taylor P. (2010). Improvement in cerebral function with treatment of posttraumatic stress disorder. Ann. N.Y. Acad. Sci. 1208, 142–149 10.1111/j.1749-6632.2010.05689.x 20955336

[B37] Salami M. Itami C. Tsumoto T. Kimura F. (2003). Change of conduction velocity by regional myelination yields constant latency irrespective of distance between thalamus and cortex. Proc. Natl. Acad. Sci. U.S.A. 100, 6174–6179 10.1073/pnas.0937380100 12719546 PMC156345

[B38] Salzman C. D. Fusi S. (2010). Emotion, cognition, and mental state representation in amygdala and prefrontal cortex. Annu. Rev. Neurosci. 33, 173–202 10.1146/annurev.neuro.051508.135256 20331363 PMC3108339

[B39] Schienle A. Schäfer A. Hermann A. Rohrmann S. Vaitl D. (2007). Symptom provocation and reduction in patients suffering from spider phobia: an fMRI study on exposure therapy. Eur. Arch. Psychiatry Clin. Neurosci. 257, 486–493 10.1007/s00406-007-0754-y 17902000

[B40] Scholz J. Klein M. C. Behrens T. E. J. Johansen-Berg H. (2009). Training induces changes in white-matter architecture. Nat. Neurosci. 12, 1370–1371 10.1038/nn.2412 19820707 PMC2770457

[B41] Singer W. (2009). Distributed processing and temporal codes in neuronal networks. Cogn. Neurodyn. 3, 189–196 10.1007/s11571-009-9087-z 19562517 PMC2727167

[B42] Stetz M. C. Thomas M. L. Russo M. B. Stetz T. A. Wildzunas R. M. McDonald J. J. (2007). Stress, mental health, and cognition: a brief review of relationships and countermeasures. Aviat. Space Environ. Med. 78(Suppl. 1), B252–B260 17547326

[B43] Straube T. Glauer M. Dilger S. Mentzel H. J. Miltner W. H. R. (2006). Effects of cognitive-behavioral therapy on brain activation in specific phobia. Neuroimage 29, 125–135 10.1016/j.neuroimage.2005.07.007 16087353

[B44] Szeligo F. Leblond C. P. (1977). Response of the three main types of glial cells of cortex and corpus callosum in rats handled during suckling or exposed to enriched, control and impoverished environments following weaning. J. Comp. Neurol. 172, 247–263 10.1002/cne.901720205 838881

[B45] Taubert M. Draganski B. Anwander A. Müller K. Horstmann A. Villringer A. (2010). Dynamic properties of human brain structure: learning-related changes in cortical areas and associated fiber connections. J. Neurosci. 30, 11670–11677 10.1523/JNEUROSCI.2567-10.2010 20810887 PMC6633410

[B46] Todd R. M. Evans J. W. Morris D. Lewis M. D. Taylor M. J. (2011). The changing face of emotion: age-related patterns of amygdala activation to salient faces. Soc. Cogn. Affect. Neurosci. 6, 12–23 10.1093/scan/nsq007 20194512 PMC3023079

[B47] Tryon W. W. (2005). Possible mechanisms for why desensitization and exposure therapy work. Clin. Psychol. Rev. 25, 67–95 10.1016/j.cpr.2004.08.005 15596081

[B48] Tryon W. W. Misurell J. R. (2008). Dissonance induction and reduction: a possible principle and connectionist mechanism for why therapies are effective. Clin. Psychol. Rev. 28, 1297–1309 10.1016/j.cpr.2008.06.003 18687510

[B49] Yamazaki Y. Hozumi Y. Kaneko K. Fujii S. Goto K. Kato H. (2010). Oligodendrocytes: facilitating axonal conduction by more than myelination. Neuroscientist 16, 11–18 10.1177/1073858409334425 19429890

[B50] Zatorre R. J. Fields R. D. Johansen-Berg H. (2012). Plasticity in gray and white: neuroimaging changes in brain structure during learning. Nat. Neurosci. 15, 528–536 10.1038/nn.3045 22426254 PMC3660656

